# Analytical Performance of the Avida Duo Assay for Simultaneous Mutation and Methylation Profiling in Circulating Cell-Free DNA

**DOI:** 10.3390/cancers18132022

**Published:** 2026-06-23

**Authors:** Russell J. Diefenbach, Ashleigh Stewart, Wei Yen Chan, Suzanah C. Boyd, Alexander M. Menzies, Georgina V. Long, Helen Rizos

**Affiliations:** 1 Macquarie Medical School, Faculty of Medicine, Health and Human Sciences, Macquarie University, Sydney 2109, Australia; russell.diefenbach@mq.edu.au (R.J.D.); ashleigh.stewart@mq.edu.au (A.S.); weiyen.chan@hdr.mq.edu.au (W.Y.C.); suzanah.boyd@hdr.mq.edu.au (S.C.B.); 2 Melanoma Institute Australia, The University of Sydney, Sydney 2065, Australia; alexander.menzies@sydney.edu.au (A.M.M.); georgina.long@sydney.edu.au (G.V.L.); 3 Faculty of Medicine and Health, The University of Sydney, Sydney 2050, Australia; 4 Department of Medical Oncology, Northern Sydney Cancer Centre, Royal North Shore Hospital, Sydney 2065, Australia; 5 Mater Hospital, Sydney 2065, Australia; 6 Charles Perkins Centre, The University of Sydney, Sydney 2050, Australia

**Keywords:** cfDNA, ctDNA, melanoma, mutation, methylation

## Abstract

**Background**: Circulating cell-free DNA (cfDNA) enables minimally invasive tumour genomic profiling, yet simultaneous interrogation of mutations and DNA methylation remains limited by assay complexity and input constraints. **Methods**: Here, we evaluate the Agilent Avida Duo system, a single workflow integrating high-sensitivity mutation detection with targeted DNA methylation analysis. We analysed 21 stage III and IV melanoma patient samples. **Results**: The Avida Duo mutation assay detected mutant allele frequencies (MAFs) down to 0.05% and identified tumour-associated mutations in all melanoma patients, including within GC-rich regions such as the *TERT* promoter. Optimisation of the Avida Duo mutation workflow, using TapeStation-quantified cfDNA and reduced amplification cycles, improved library consistency without compromising sensitivity. Methylation profiling of the melanoma baseline cohort with the Avida Duo methylation panel showed high concordance with QIAseq targeted methylation results, with the mean cfDNA fraction methylation ranging from 0.051–0.079 in most patients and reaching 0.249 in the patient with the highest ctDNA burden (MAFs up to 35.4%). **Conclusions**: Our findings demonstrate that the Avida Duo workflow enables simultaneous, high-resolution detection of mutation and methylation profiles from a single cfDNA sample, streamlining processing and enhancing molecular insight for clinical and translational applications.

## Simple Summary

In the field of liquid biopsy, assessing the mutation and methylation changes in circulating free DNA has become increasingly important for cancer detection and treatment selection. The Avida Duo assay from Agilent represents a highly streamlined workflow incorporating dual mutation and methylation analysis. In this study, we have tested the Avida Duo assay using plasma circulating free DNA from two independent cohorts of patients with advanced melanoma. We optimised the Avida workflow to improve the consistency of assay output and maximise the sensitivity of mutation detection. We confirm that Avida Duo methylation detection has high concordance with Qiagen’s QIAseq targeted methylation detection. Collectively, these results demonstrate that the Avida Duo assay provides a highly sensitive and robust method for cancer detection, monitoring and treatment guidance.

## 1. Introduction

Analysis of circulating free DNA (cfDNA) has reshaped cancer prediction and prognostic assessment by enabling minimally invasive sampling. Multimodal approaches for detecting circulating tumour DNA (ctDNA), the cancer-derived fraction of cfDNA, improve sensitivity of detection and support treatment response monitoring and prognostication [1,2]. These approaches interrogate mutation, methylation and fragmentomic features, but are typically performed using separate assays [3,4]. Given the limited quantity of cfDNA in many clinical samples, there is growing interest in developing integrated mutation–methylation assays that maximise analytical sensitivity while reducing input requirements [5,6,7,8,9].

Methylation of cfDNA has emerged as a promising biomarker for cancer detection, given that methylation changes typically appear early in cancer progression [10] and may provide a sensitive signal when tumour-derived mutations are present at low frequency. Combining mutation and methylation analysis of cfDNA has been shown to improve the sensitivity of cancer detection, particularly in early-stage and minimal residual disease settings [6,11]. Circulating cfDNA levels decrease with lower tumour burden, and in melanoma, ctDNA detection rates based on mutation analysis range from 47% in stage III to 84% in stage IV disease [12]. Accordingly, a dual mutation and methylation assay may enhance overall analytical sensitivity and robustness when cfDNA input is limited, particularly in early-stage melanoma.

The Avida Duo assay (Agilent) [13,14] is among the first commercially available platforms enabling the simultaneous detection of cfDNA mutation and methylation from a single cfDNA input. The 5-Base DNA Prep with Enrichment assay (Illumina) for the simultaneous detection of cfDNA mutation and methylation in the same workflow is now also available. In this study, we evaluated the performance of the Avida Duo assay in stage III and IV melanoma samples. Overall, the Avida Duo assay detected melanoma-associated mutations in all patients and demonstrated a high degree of correlation with Qiagen’s QIAseq targeted methylation workflow.

## 2. Materials and Methods

### 2.1. Human Melanoma Samples

Fresh-frozen tissue and blood samples from melanoma patients were obtained from the Melanoma Institute Australia biospecimen bank with written informed patient consent and institutional review board approval (Sydney Local Health District Human Research Ethics Committee, Protocol No. X15–0454 and HREC/11/RPAH/444). Oncofocus/OncoCarta panels v1.0 (Agena Bioscience, San Diego, CA, USA) were used for detection of melanoma-associated *BRAF*, *NRAS*, *KRAS* and *KIT* variants in tissue samples [15]. Immunohistochemistry to detect *BRAF* V600E using VE1 monoclonal antibody (Abcam, Cambridge, UK) was performed as previously described [16].

Blood (10 mL) was collected in EDTA tubes (Becton Dickinson, Franklin Lakes, NJ, USA) and processed within 4 h from blood draw. Tubes were spun at 800× *g* for 15 min at room temperature. Plasma was then removed into new 15 mL tubes without disturbing the buffy coat and respun at 1600× *g* for 10 min at room temperature to remove cellular debris. Plasma was stored in 1–2 mL aliquots at −80 °C, and thawed only once to preserve cfDNA quality [17].

### 2.2. Purification of cfDNA from Plasma

cfDNA was extracted from plasma using the QIAamp Circulating Nucleic Acid Kit (Qiagen, Hilden, Germany). Samples with 4 mL or less of plasma were processed as per manufacturer’s instructions. Samples with more than 4 mL of plasma were made up to 8 mL with PBS and then divided into 4 mL volumes and processed separately before combining on one QIAamp mini column. To increase cfDNA yield, all samples were re-eluted using the initial 100 μL of dH_2_O. cfDNA was quantified using the Qubit High Sensitivity dsDNA Kit and the Qubit 3.0 Fluorometer (Thermo Fisher Scientific, Waltham, MA, USA). cfDNA purity, 100–700 bp range, was determined using a Cell-free DNA ScreenTape and TapeStation 4150 (Agilent Technologies, Santa Clara, CA, USA).

### 2.3. Preparation of Avida Duo Libraries

The generation of Avida Duo libraries, including library preparation, target capture, conversion, and indexing was performed using the Avida Duo reagent kit (Agilent Technologies, Santa Clara, CA, USA) according to the manufacturer’s instructions. Targeted panels used for sequencing included the Avida DNA expanded cancer mutation panel, which is predicted to cover 92% of cutaneous melanoma patients based on interrogation of 1936 patients in https://www.cbioportal.org [18,19,20]; and the Avida methyl 3400 DMR cancer panel, which covers 3482 differentially methylated regions associated with cancer. In addition, we included Seraseq ctDNA Mutation Mix v4 AF 0.5% (Seracare, Milford, MA, USA) as a positive control.

The Avida DNA expanded cancer mutation panel includes the following gene targets: *ABL1*, *AKT1*, *AKT2*, *ALK*, *AR*, *ARAF*, *ARID1A*, *ATM*, *ATR*, *ATRX*, *B2M*, *BAP1*, *BARD1*, *BRAF*, *BRCA1*, *BRCA2*, *BRD4*, *BRIP1*, *CCND1*, *CD274*, *CDK12*, *CDK4*, *CDKN2A*, *CHEK1*, *CHEK2*, *CTNNB1*, *DDR2*, *DNMT3A*, *EGFR*, *ERBB2*, *ERBB3*, *ERBB4*, *ERCC2*, *ESR1*, *EZH2*, *FANCA*, *FANCC*, *FANCL*, *FBXW7*, *FGF3*, *FGFR1*, *FGFR2*, *FGFR3*, *FGFR4*, *FLT3*, *GNA11*, *GNAQ*, *GNAS*, *HGF*, *HRAS*, *IDH1*, *IDH2*, *JAK1*, *JAK2*, *KEAP1*, *KIT*, *KRAS*, *MAP2K1*, *MAP2K2*, *MDM2*, *MET*, *MLH1*, *MSH2*, *MSH6*, *MTOR*, *MYC*, *MYD88*, *NF1*, *NF2*, *NPM1*, *NRAS*, *NTRK1*, *NTRK2*, *NTRK3*, *PALB2*, *PDGFRA*, *PDGFRB*, *PIK3CA*, *PMS2*, *POLE*, *PTCH1*, *PTEN*, *PTPN11*, *RAC1*, *RAD50*, *RAD51B*, *RAD51C*, *RAD51D*, *RAD54L*, *RAF1*, *RB1*, *RET*, *RICTOR*, *ROS1*, *SMARCA4*, *SMARCB1*, *SMO*, *STAG2*, *STK11*, *TERT* promoter, *TP53*, *TSC1*, *TSC2*, *VHL*.

The Avida workflow was performed over two days for the Duo workflow, or one day if only performing mutation detection, and consists of the following steps:

Day 1

- PCR-free library preparation.
- First target capture for targeted sequencing (DNA expanded cancer mutation panel).
- Supernatant from first target capture collected for second target capture.
- Second target capture hybridisation performed overnight.
- Indexing PCR for targeted sequencing (store overnight at 4 °C).
  ○ Note: if only performing targeted sequencing, continue with library purification and library quantification on day 1.

Day *2*

- Finish second target capture for methyl sequencing (methyl 3400 DMR cancer panel).
- Soft bisulfite conversion.
- Indexing PCR for methyl sequencing.
- Library purification of targeted and methyl sequencing library samples.

Libraries were subsequently checked for quality and quantified using D1000 ScreenTape and TapeStation 4150 (Agilent Technologies, Santa Clara, CA, USA). Final Avida Duo libraries were stored at −30 °C.

### 2.4. Sequencing of Avida Duo Libraries

Sequencing libraries were pooled in low TE buffer. Starting library concentrations were based on D1000 TapeStation determination (200–1000 bp). Dilution calculations were based on an allocation of 80 million paired end (PE) reads for the Avida DNA expanded cancer panel and 10 million PE reads for the Avida methyl 3400 DMR cancer panel. Pooled library quality control and sequencing was performed by the Australian Genome Research Facility (AGRF, Melbourne, Victoria, Australia). The final pooled library concentration used for sequencing was 160 pM and included 2% PhiX. Sequencing (150 bp PEs) was performed using one lane of a Novaseq X 10B flow cell (Illumina, San Diego, CA, USA) run on a Novaseq X instrument (Illumina, San Diego, CA, USA).

### 2.5. Analysis of Avida Duo Sequencing

Sequencing data were processed based on the Avida DNA Targeted Sequence Analysis Technical Guide (Version B1, December 2024). Raw FASTQ files were quality assessed using FastQC (v0.12.1) and summarised with MultiQC (v1.14). Reads were converted to unmapped BAM format using fgbio (v2.2.1) with defined read structures (3M2S+T) to extract unique molecular identifiers (UMIs). Alignment to the GRCh38 reference genome was performed using BWA-MEM (v0.7.17) [21], and BAM processing utilised SAMtools (v1.6). Duplication rates were determined using Picard MarkDuplicates. UMI family size distributions were derived from fgbio GroupReadsByUmi using the family-size histogram output. Duplex and molecular consensus reads were generated using fgbio with the following filtering parameters: minimum input base quality ≥ 10–20, minimum of 2 reads per consensus family, and stringent post-consensus base quality and error-rate thresholds (minimum base quality ≥ 45 and maximum base error rate ≤ 0.2). Mutation calling was performed using VarDict (2019.06.04; VarDict-java v1.8.3) [22], followed by strand bias filtering. For VarDict variant calling, the minimum number of variants supporting reads was set to 4 for molecular consensus and 2 for duplex consensus. Targeted coverage metrics were calculated using Picard tools and related genome analysis toolkit (GATK v4.3.0.0) [23] utilities. All software versions were recorded to ensure reproducibility. Final mutation annotation was performed using Qiagen clinical insight (Qiagen, Hilden, Germany).

Methylation calling workflow was based on technical guide G9419-90001 (Agilent Technologies, Santa Clara, CA, USA). Specific open source software packages employed included Bismark (v0.25.1) [24], Bowtie [25] and SAMtools (v1.6) [26].

### 2.6. Preparation of QIAseq Targeted Methylation Libraries

A made-to-order QIAseq targeted methylation panel (5000 primers) was obtained from Qiagen (Hilden, Germany). The QIAseq targeted methylation panel workflow including bisulfite conversion was performed according to the manufacturer’s instructions with the exception that QIAseq beads at a 1.2× ratio were used in all clean-up steps. For library amplification, the number of cycles was set to 19.

Library quality control and sequencing were performed by AGRF (Melbourne, Victoria, Australia). Library concentrations were calculated based on the median size of the library and subsequently pooled to 4 nM. The final library concentration used for sequencing was 140 pM and included 10% PhiX. Sequencing primer QIAseq A Read1 Custom Primer I (Qiagen, Hilden, Germany) was used according to the manufacturer’s instructions. Sequencing (150 bp PEs) was performed using one lane of a Novaseq X 10B flow cell (Illumina, San Diego, CA, USA) run on a Novaseq X instrument (Illumina, San Diego, CA, USA). Analysis of FASTQ sequencing files to determine fraction methylation at individual CpG sites was performed using CLC Genomics Workbench version 23.03 (Qiagen, Hilden, Germany).

### 2.7. Droplet Digital PCR (ddPCR)

Preamplification followed by ddPCR for detection of *BRAF* V600E and *NRAS* Q61R was performed as previously described [27,28]. Briefly, the DNA TOP-PCR cfDNA pre-amplification kit (Top Science Biotechnologies, Taiwan, Cat No. D01) was used to enhance ctDNA sensitivity [29]. Ligation and amplification were performed as described by the manufacturer, except that input cfDNA was 20 ng and amplification was for 5 cycles. Subsequently, 5 µL of pre-amplified cfDNA (10–40 ng) underwent three independent ddPCR experiments to validate ctDNA positivity. For ddPCR, ctDNA was amplified in 22 µL reactions using the BioRad QX600 AutoDG ddPCR system (BioRad, Hercules, CA, USA) and BioRad primer/probes (BioRad, Hercules, CA, USA) for specific mutations.

### 2.8. Statistical Analysis

Spearman’s and Mann–Whitney tests were performed using Graphpad Prism v 11.

## 3. Results

### 3.1. Limited cfDNA Yield in Early-Stage Melanoma Supports Integrated Profiling Approaches

To illustrate the value of an integrated mutation–methylation assay, we analysed plasma cfDNA yields from several historic melanoma cohorts within our liquid biopsy program (Figure S1). In stage III patients, median cfDNA yield was 9.7 ng/mL plasma, whereas stage IV patients showed a broader range and higher median yield (range: 1.85–133.0 ng/mL, median: 11.8 ng/mL plasma; Mann–Whitney, *p* < 0.01). These data underscore the need for integrated approaches that enable cfDNA mutation and methylation analyses from limited cfDNA input.

### 3.2. Cohort 1 and Sample Characteristics

A total of 14 patients with cutaneous melanoma and plasma collected at baseline (PRE) were included in cohort 1 (Table 1). Of these, 9/14 (64%) had stage III pre-operative disease (median age 68 years) and 5/14 (36%) had stage IV disease (median age 73 years). The majority of patients (9/14 (64%)) were male. Tumour tissue mutation data were available for all patients: 6/14 (43%) had a *BRAF* mutation, 4/14 (29%) had an *NRAS* mutation, 3/14 (21%) were wild-type for hotspot mutations in *BRAF* and one patient (7%) was wild-type for hotspot mutations in *BRAF*, *NRAS* and *KIT*. Cohort 1 stage IV patients had a higher cfDNA yield than stage III patients.

### 3.3. Avida Duo Library Yield and Avida Mutation Panel Performance

**Figure 1:**
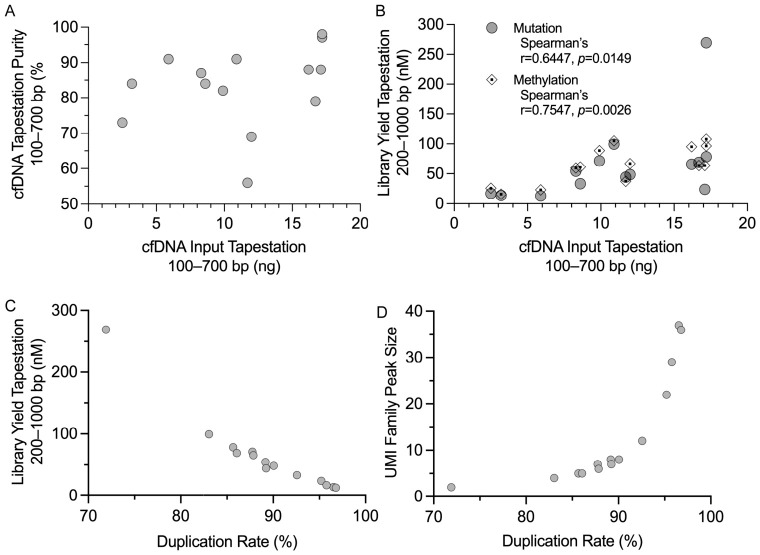
Cohort 1 input cfDNA and library complexity. Input cfDNA versus either cfDNA purity (**A**) or library concentration for Avida Duo mutation and methylation panels (**B**). Input cfDNA and purity was based on TapeStation (cfDNA screentape; 100–700 bp). Avida mutation panel library complexity based on duplication rate versus either library concentration (**C**) or UMI family peak size (**D**). Library concentration was based on TapeStation (D1000 screentape; 200–1000 bp range).

For cohort 1, 20 ng of cfDNA (Qubit-based quantification) was used in the Avida Duo workflow. However, Qubit-based quantification overestimated cfDNA input across samples. Although cfDNA samples were normalised to 20 ng by Qubit, fragment analysis by TapeStation indicated a 100–700 bp input range of 2.5–17.2 ng DNA, with variable DNA purity (56–98%) (Figure 1A). Library yield varied across samples and correlated more strongly with TapeStation-derived cfDNA than Qubit estimates (Figure 1B), indicating that effective cfDNA input was the primary determinant of library performance.

Most cohort 1 samples achieved the expected sequencing output (~80 million PE reads, range 66–94 million PE reads) with the Avida mutation panel, although library complexity varied substantially (Figure 1C,D). One exception was patient sample 8, which, despite a lower-than-expected library yield, achieved a high read depth of 176 million PE reads. Duplication rates were high overall (72–97%) and were inversely related to library concentration and complexity, as reflected by UMI family peak size distributions (Figure 1C,D). Higher-complexity libraries improved sequencing efficiency and coverage, whereas lower complexity samples exhibited reduced effective depth despite comparable sequencing output (Figure 1C,D).

### 3.4. Molecular and Duplex Consensus Coverage Reflect Library Complexity

**Figure 2:**
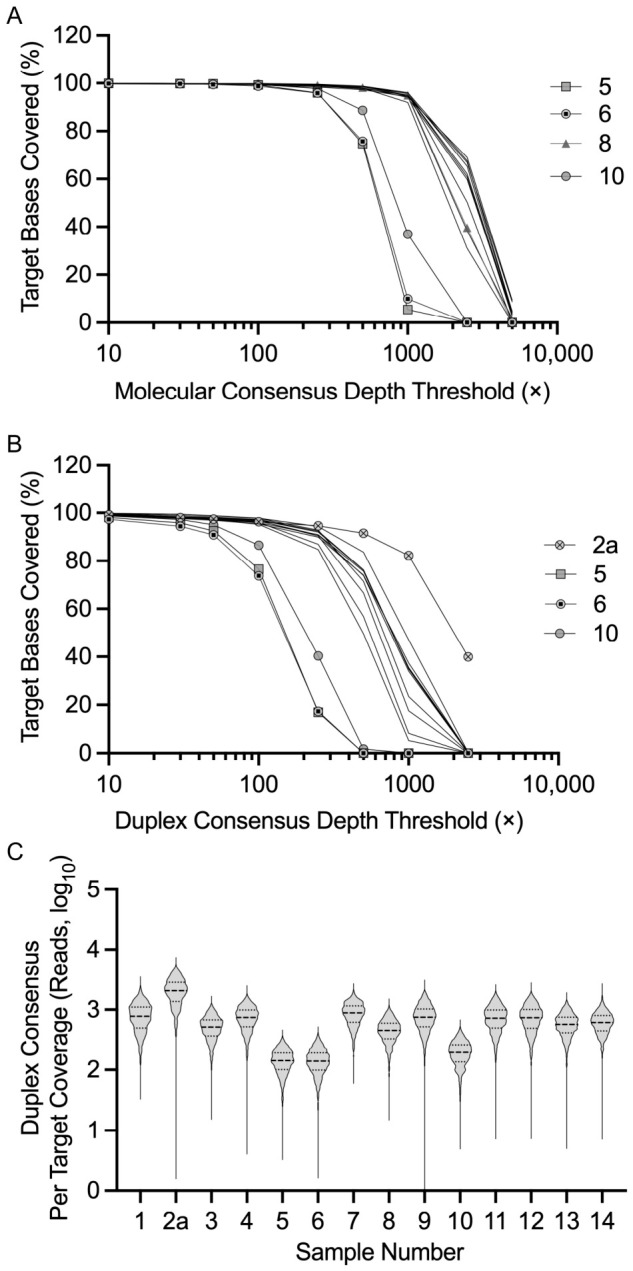
Cohort 1 target coverage for the Avida mutation panel. Percentage target bases covered at increasing depth thresholds was determined based on molecular (**A**) or duplex (**B**) consensus. (**C**) Per-target coverage based on duplex consensus. Dark dashed line represents median, light dashed line represent 25th and 75th percentile respectively.

Molecular consensus coverage was maintained across all cohort 1 samples at low depth thresholds (10×–100×). Notably, samples 5, 6 and 10 showed a rapid loss of coverage, with near-complete depletion at ≥1000×, consistent with lower library concentrations and limited molecular complexity (Figure 2A). In contrast, despite generating the highest number of PE reads, sample 8 did not show improved high-threshold coverage (Figure 2A), demonstrating that sequencing depth alone is insufficient for limited library complexity.

Duplex consensus coverage was substantially reduced relative to molecular consensus across all samples, reflecting the requirement for complementary strand support for duplex reconstruction (Figure 2B). Sample 2a was the only sample with appreciable duplex recovery at ≥1000×, consistent with its high library complexity. In contrast, samples 5, 6 and 10 showed minimal duplex support, in line with their limited molecular diversity.

Per-target coverage varied across genomic regions but showed highly consistent patterns between samples (Figure 2C). A subset of regions, including *RB1* exon 15, *POLE* hotspot regions, and *BRCA2* exon 11, exhibited systematically lower coverage across the samples (Table S1). The structurally challenging *TERT* promoter region also showed reduced coverage, consistent with its high GC content and repetitive sequence (Table S1). Importantly, these patterns were consistent across samples, indicating that variability in overall coverage was primarily driven by library complexity rather than target-specific effects. Accordingly, higher-complexity samples (e.g., 2a, 7) demonstrated increased coverage across targets, whereas low-complexity samples (e.g., 5, 6, 10) showed consistently reduced coverage across all targets.

### 3.5. Performance Evaluation with the Seraseq ctDNA Reference Standard

To assess Avida mutation panel performance, 20 ng of a Seraseq ctDNA reference control was included. The Seraseq library exhibited moderate molecular complexity (duplication 85%, UMI family peak size 5, 8.2 × 10^7^ PE reads), comparable to mid-range patient samples. Duplex consensus coverage declined to 54% at 1000×, consistent with the expected behaviour of libraries with modest complementary strand recovery.

All 59 Seraseq ctDNA somatic mutations covered by the Avida mutation panel were detected. Molecular consensus identified 98% (58/59) variants while duplex consensus identified 95% (56/59) mutations (Figure S2). Mutations detected only by molecular consensus included *AKT1* E17K, *AR* H875Y and *IDH1* R132C, while the mutation *RB1* R251* was only detected by duplex consensus (Table S1 includes all mutations detected in the Seraseq control).

### 3.6. Avida Mutation Panel Patient Results

**Figure 3:**
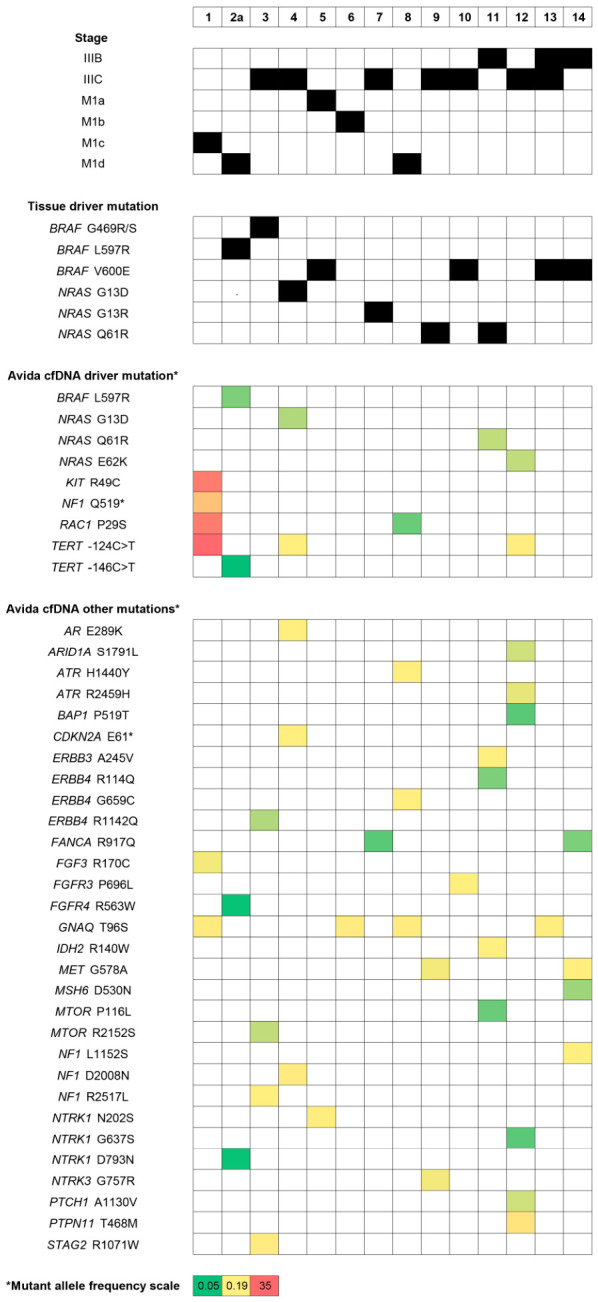
Summary of cohort 1 and mutations identified by the Avida mutation panel. Avida cfDNA mutations were confirmed to be melanoma-associated using cbioportal (https://www.cbioportal.org).

Across cohort 1, cfDNA mutations were identified in all samples using the Avida mutation panel (Table S1). Nonsynonymous mutations in *ATM*, *CHEK2*, *DNMT3A* and *TP53* were identified across multiple samples (Table S1) and were excluded from downstream interpretation as likely arising from clonal haematopoiesis of indeterminate potential (CHIP) [30,31]. Ultimately gDNA sequencing using patient-matched PBMCs would be required to eliminate all potential CHIP variants. Of the remaining nonsynomous somatic mutations (excluding CHIP mutations) considered to be melanoma-associated (based on cbioportal), 67% (31/46) were detected by both molecular and duplex consensus, 11% (5/46) by molecular consensus only and 22% (10/46) by duplex consensus only (Figure 3). Detection sensitivity reached 0.09% mutant allele frequency (MAF) for molecular consensus and 0.05% MAF for duplex consensus calling.

Nine patients had known tissue driver mutations covered by the Avida mutation panel. Of these, three samples (2a, 4, 11) demonstrated concordant detection of driver mutations in both tumour tissue and plasma cfDNA (Figure 3). These samples included one stage III patient (sample 4), spanned a broad range of cfDNA input (9.9–17.2 ng by Tape-Station), and exhibited variable library complexity (UMI family peak size 2–8), with all achieving >65% coverage at 2500×. Samples 2a and 4 also harboured *TERT* promoter driver mutations identified by Avida sequencing. In addition to canonical drivers, these three samples contained additional melanoma-associated mutations (MAF <1%), including putative driver alterations *NF1* D2008N (sample 2a), *CDKN2A* E61* (sample 4) and *ERBB4* R114Q (sample 11). In sample 4, multiple mutations, including *NRAS* G13D, *CDKN2A* E61*, *NF1* D2008N and *TERT* promoter mutations, were detected by the Avida mutation panel (Figure 3).

Three stage III samples (7, 9, 13) had similar sequencing outputs, but the tissue driver mutations were not detected in the circulation by Avida, and, in the case of samples 9 and 13, nor by ddPCR, suggesting limited tumour shedding rather than insufficient sequencing performance. Nevertheless, Avida identified low allele frequency mutations in these three samples (Figure 3). The final three samples (5, 10 and 14) had insufficient depth for reliable detection of low-frequency mutations with molecular depth falling below 40% at 2500×. Sample 10 was also subjected to ddPCR, which also failed to detect the driver mutation. Across these six samples (5, 7, 9, 10, 13 and 14), several additional melanoma-associated mutations were identified at low MAFs (<1%).

The remaining five patients, based on tissue screening, either had no known driver mutation, or, in the case of sample 3, had a *BRAF* G469R/S driver mutation not covered by the Avida mutation panel (Figure 3). Samples 1, 8 and 12, which exhibited moderate molecular depth (40–70% at 2500×), all had driver mutations identified by the Avida mutation panel. In sample 1 (stage M1c), multiple driver mutations, including *RAC1* P29S and *TERT* -124C>T, were detected at high MAFs (>10%). Samples 3 and 6, with low molecular depth < 32% at 2500×, had melanoma-associated mutations detected at low allele frequencies (MAF < 3%, with majority < 1%). Putative driver mutations included *ERBB4* G659C (sample 8), *ERBB4* R1142Q (sample 3), *NF1* R2517L (sample 3) and *PTPN11* T468M (sample 12).

### 3.7. Optimising the Avida Mutation Panel Workflow

Initial Avida Duo assay trials used 20 ng cfDNA input based on Qubit quantification. The workflow was subsequently optimised by increasing cfDNA input to 40 ng and requiring > 80% purity, both assessed by TapeStation. In addition, the number of amplification cycles in the final indexing step was reduced from 14 to 12.

**Figure 4:**
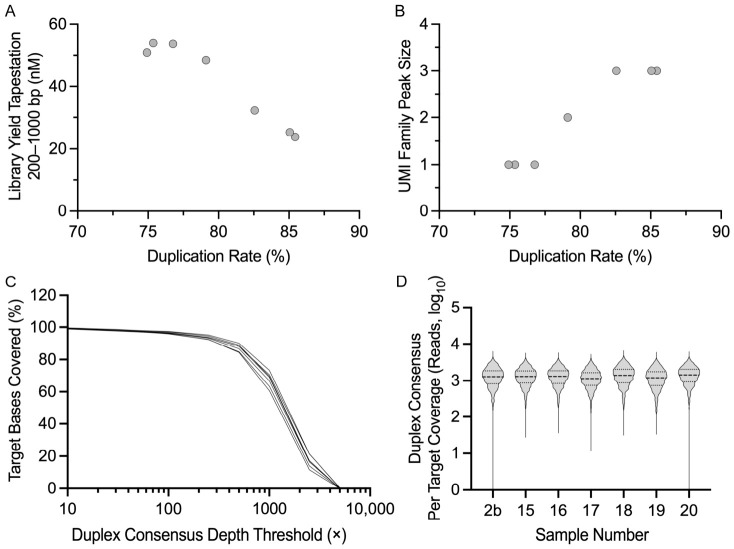
Library complexity and target coverage for cohort 2 based on the Avida mutation panel. Duplication rate versus either library concentration (**A**) or UMI family peak size (**B**). (**C**) Percentage target bases covered at increasing depth thresholds. (**D**) Per-target coverage. Dark dashed line represents median, light dashed line represent 25th and 75th percentile respectively. Library concentration was based on TapeStation (D1000 screentape; 200–1000 bp range).

Data from seven patients (Table 1, cohort 2) sampled early during treatment (EDT; 5–11 weeks on immunotherapy), demonstrates the performance of the optimised workflow. All samples achieved the expected sequencing output of ~80 million PE reads (range of 74–105 million). The combination of reduced amplification cycles and increased Tape-Station-verified cfDNA input consistently decreased duplication rates, reduced variability in library concentration, and increased library complexity, as reflected by lower peak UMI family sizes (Figure 4A,B) compared with the initial workflow. Increased cfDNA input also resulted in consistent depth of coverage across all samples, as well as uniform target coverage across the panel (Figure 4C,D). Target coverage is summarised in Table S2. Importantly, overall target coverage was comparable to that observed in cohort 1 using the initial workflow.

**Figure 5:**
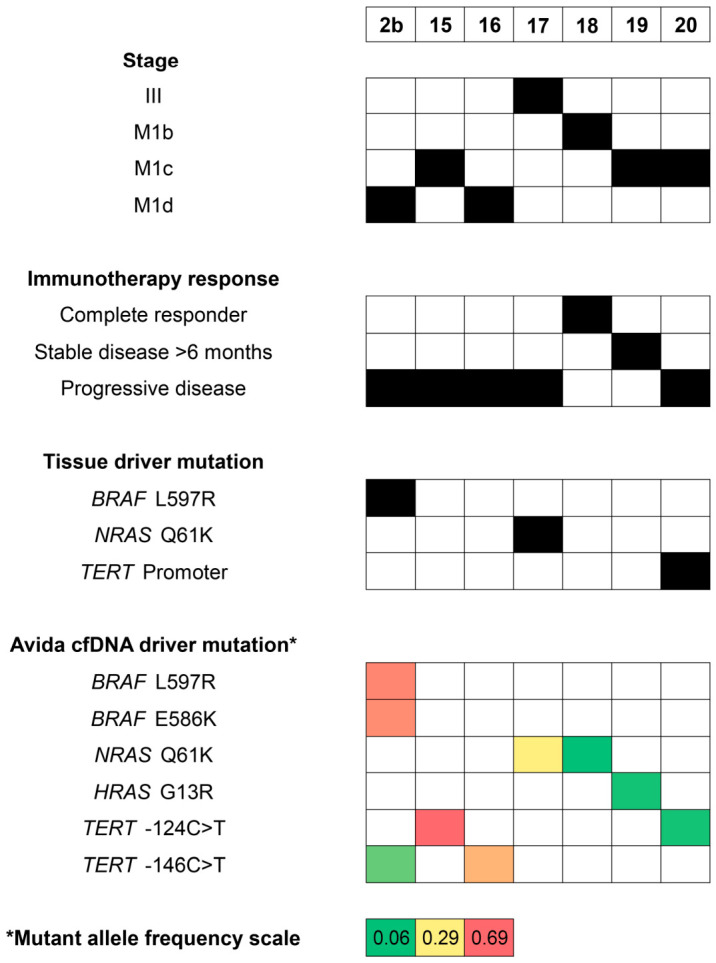
Summary of cohort 2 and mutations identified by the Avida mutation panel. Avida cfDNA mutations were confirmed to be melanoma-associated using cbioportal (https://www.cbioportal.org). Immunotherapy consisted of either pembrolizumab alone or combined ipilimumab and nivolumab.

Mutation calling for these seven melanoma EDT samples identified driver mutations in all cases (Figure 5). Due to the limited sample size of this pilot cohort (*n* = 2 responders and *n* = 5 non-responders), statistical evaluation of clinicopathological associations was not feasible. Descriptively, however, total cfDNA yields appeared to track higher in the majority of non-responding patients (yield range: 15.8–36.9 ng/mL plasma) compared to responding patients (yield range: 11.8–13.4 ng/mL plasma). These observations remain strictly exploratory until validated in a larger, adequately powered cohort.

Driver mutations included *TERT* promoter c.-146C>T and c.-124C>T, *NRAS* Q61K, *HRAS* G13R, *BRAF* L597R and *BRAF* E586K, which ranged in MAF from 0.06% to 0.69%. Aside from the one *NRAS* Q61K mutation with a MAF of 0.06%, all were identified using molecular and duplex consensus workflows. The three patients with known tissue mutations were all identified by the Avida mutation panel. Sample 2 was included in both cohort 1 (sample 2a, PRE) and cohort 2 (sample 2b, EDT). This patient, a non-responder to immunotherapy, had, at both PRE and EDT, a *BRAF* L597R mutation (MAF increased from 0.1 to 0.6%) and a *TERT* promoter c.-146C>T mutation (MAF increased from 0.05 to 0.12%). In addition, at EDT, a *BRAF* E586K mutation (MAF 0.58%) was present in sample 2b. All identified mutations for cohort 2 are summarised in Table S2.

### 3.8. Avida Methylation Panel Performance

For the Avida methylation panel, the PE read output for 13 of 14 samples from cohort 1 ranged from 8–11 million PE reads, consistent with the expected yield of ~10 million PE reads based on library input. The exception was sample 2a, which generated 15 million PE reads despite having a methyl library concentration similar to those of the other samples. This sample also produced high mutation panel read depth and low duplication in the Avida DNA expanded cancer panel, consistent with its overall higher molecular complexity across assays.

**Figure 6:**
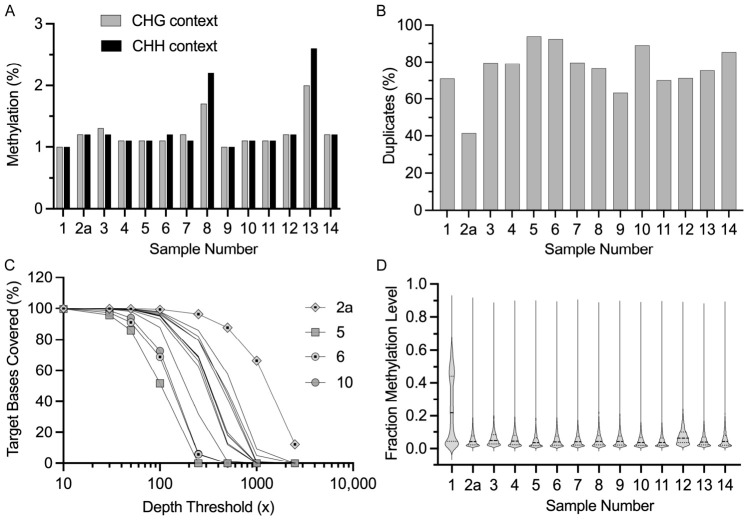
Performance of the Avida methylation panel based on cohort 1. (**A**) Efficiency of bisulfite conversion. (**B**) Sequence duplication rates. (**C**) Percentage target bases covered at increasing depth thresholds. (**D**) Distribution of fraction methylation levels across samples. Dark dashed line represents median, light dashed line represent 25th and 75th percentile respectively. CHG or CHH context (H corresponds to A, T or C).

Methylated cytosines in non-CpG contexts (CHG and CHH, where H = A, C or T) occur at very low levels in most mammalian cells [32]. Bisulfite conversion efficiency was high, as expected. Based on CHG context, efficiency ranged from 98–99%, and, based on CHH context, ranged from 97.4–99% (Figure 6A). The lowest conversion efficiencies, i.e., the highest residual methylation in CHG/CHH contexts, were observed in samples 8 and 13 (Figure 6A).

For 13 of 14 samples, final duplicate levels ranged from 63–94%, with patient 2a showing the lowest duplication rate (41%) and, correspondingly, the highest coverage depth (Figure 6B,C). In contrast, patient samples 5, 6 and 10, with the lowest methylation panel library concentrations, exhibited the highest duplication rates and the lowest coverage depth (Figure 6B,C).

The fraction methylation level for each region encompassed by the Avida methylation panel was determined for each sample (Table S3). For 13/14 patient samples, the mean methylation level ranged from 0.051 to 0.079 (Figure 6D). The exception was patient sample 1, which had a mean methylation level of 0.249 (Figure 6D). Based on variant analysis, this sample had the highest ctDNA burden, with a *TERT* promoter c.-124C>T mutation (MAF 35.4%), a *RAC1* P29S mutation (MAF 30.5%) and a *NF1* Q519* mutation (MAF 11.6%) (Figure 3). All the other samples had mutations at much lower MAFs (Figure 3). Across all samples, the worst-performing methylation target region for coverage was chr1:148808737-148808918 (Table S3).

### 3.9. Comparison of Avida and Qiagen Methylation Workflows

**Figure 7:**
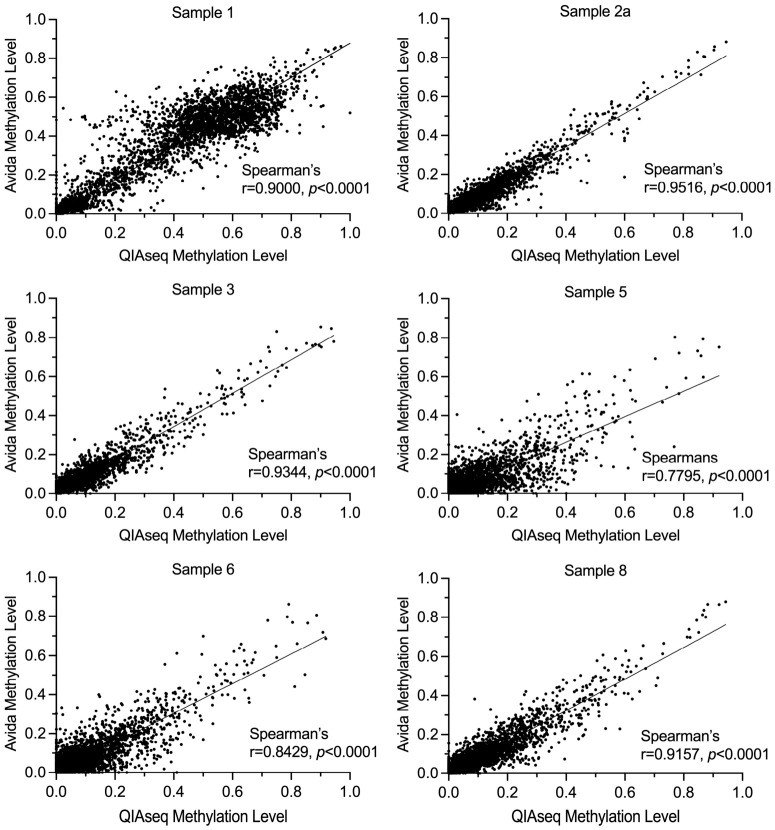
Correlation of Avida and QIAseq methylation workflows based on selected cohort 1 samples. Correlation was based on CpG sites present in both panels.

Of the 14 cohort 1 baseline samples processed using the Avida methylation panel, six samples had previously been analysed using a Qiagen QIAseq targeted methylation panel workflow (using 20 ng Qubit-based cfDNA input). This enabled direct comparison of the two methods using fractional methylation data across 3218 CpG sites common to both panels (Table S4). Pearson correlation was significant across all samples, with the highest concordance observed in sample 2a and the lowest in sample 5 (Figure 7).

## 4. Discussion

The Avida Duo assay enables simultaneous detection of both somatic mutations and DNA methylation from cfDNA derived from a single patient sample. This approach conserves limited cfDNA input and can be completed within 1–1.5 days. The assay employs unique bridge probes that enable PCR-free hybridisation capture of both mutation and methylation targets, with amplification restricted to the final PCR-based indexing step. Within this workflow, mutation capture is performed first, followed by methylation capture using the same cfDNA template.

A limitation of the Avida approach arises when the simultaneous assessment of mutation and methylation at the same genomic site is required. Aside from one exon site in gene *ESR1*, no other overlap is present in the off-the-shelf Avida duo panels used in this study. If significant overlap is present in custom designed panels, then the nature of the Avida Duo assay—with mutation targeted capture first, followed by methylation targeted capture—could potentially impede methylation analysis. Alternative methods addressing this limitation have been reported for the codetection of mutation and methylation [5,6,7,8,9]. To our knowledge, these multiplex cfDNA assays are not yet commercially available for research. One example, the mutation capsule plus technology described by Wang et al. [6] is offered by Genetron as a patient service.

Our findings demonstrate that the Avida Duo assay provides a robust framework for simultaneous mutation and methylation profiling from limited cfDNA input, achieving sensitive detection of melanoma-associated mutations across an advanced disease cohort. The use of dual-strand UMI tagging and combined molecular and duplex consensus calling underpins the high analytical specificity observed, consistent with improved suppression of sequencing artefacts and enhanced confidence in low-frequency mutation detection [33,34,35]. In addition, the Avida Duo methylation panel showed strong concordance with a QIAseq targeted methylation assay.

Importantly, our data highlight that pre-analytical cfDNA quantification is a critical determinant of downstream performance. Reliance on Qubit-based measurements led to systematic overestimation of usable cfDNA input, adversely affecting library complexity and sequencing efficiency. Our findings support an optimised cfDNA workflow for the Avida Duo assay that improves both analytical consistency and cost efficiency. We propose the use of TapeStation-based quantification of the 100–700 bp cfDNA fraction with a standardised input of 40 ng, combined with a reduced number of indexing PCR cycles to preserve library complexity. Within this framework, sequencing to approximately 80 million PE reads for mutation analysis and 10 million PE reads for methylation analysis appears sufficient to achieve robust sensitivity while maintaining efficient resource utilisation. This streamlined approach balances depth of coverage, molecular complexity, and assay cost, and provides a practical basis for scalable clinical implementation of combined cfDNA mutation and methylation profiling.

## 5. Conclusions

In this study, we optimised the Avida Duo workflow to improve library consistency without compromising sensitivity and highlighted the value of evaluating both molecular- and duplex-consensus strategies for accurate mutation calling. In parallel, comparison of Avida Duo methylation workflow with the QIAseq targeted methylation platform (Qiagen) across shared CpG methylation sites demonstrated a high degree of correlation. The Avida Duo assay represents a robust approach for dual detection of cfDNA mutation and methylation.

## Figures and Tables

**Table 1:** Patient cohorts.

	Cohort 1 (Baseline)		Cohort 2 (EDT)
Clinical Characteristics	Stage III (*n* = 9)	Stage IV (*n* = 5)	Stage III (*n* = 1)/IV (*n* = 6)
Age—median (range)	68 (58–82)	73 (41–87)	66 (54–94)
Sex—no. (%)			
Male	6 (67)	3 (60)	5 (71)
Female	3 (33)	2 (40)	2 (29)
AJCC tumour stage—no. (%)			
IIIb	2 (22)		
IIIb/IIIc	1 (11)		
IIIc	6 (67)		1 (14)
M1a		1 (20)	0 (0)
M1b		1 (20)	1 (14)
M1c		1 (20)	3 (43)
M1d		2 (40)	2 (29)
Mutation—no. (%)			
*BRAF* V600E	3 (33)	1 (20)	0 (0)
*BRAF* G469R	1 (11)	0 (0)	0 (0)
*BRAF* L597R	0 (0)	1 (20)	1 (14)
*NRAS* Q61K/R	2 (22)	0 (0)	1 (14)
*NRAS* G13/D/R	2 (22)	0 (0)	0 (0)
*BRAF* WT	1 (22)	2 (40)	0 (0)
*BRAF*/*NRAS* WT	0 (0)	0 (0)	2 (29)
*BRAF*/*NRAS*/*KIT* WT	0 (0)	1 (20)	2 (29)
*TERT* Promoter	0 (0)	0 (0)	1 (14)
cfDNA yield (ng/mL plasma)—median (range)	18 (11–51)	71 (29–171)	17 (12–37)

Abbreviations: AJCC, American Joint Committee on Cancer; EDT, early during treatment (5–11 weeks from immunotherapy initiation).

## Data Availability

The raw data supporting the conclusions of this article will be made available by the authors on request.

## Supplementary Materials

The following supporting information can be downloaded at: https://www.mdpi.com/article/10.3390/cancers18132022/s1, Figure S1: Melanoma cfDNA yields; Figure S2: Seraseq ctDNA control; Table S1: Cohort 1 target coverage and mutations; Table S2: Cohort 2 target coverage and mutations; Table S3: Cohort 1 methylation; Table S4: Cohort 1 methylation workflow comparison.

## Abbreviations

The following abbreviations are used in this manuscript:

AGRF	Australian Genome Research Facility
cfDNA	Circulating free DNA
CHIP	Clonal haematopoiesis of indeterminate potential
ctDNA	Circulating tumour DNA
ddPCR	Droplet digital PCR
EDT	Early during treatment
GATK	Genome analysis toolkit
MAF	Mutant allele frequency
PE	Paired end
UMI	Unique molecular identifier
