# Supplementary figures and images for: Analytical Performance of the Avida Duo Assay for Simultaneous Mutation and Methylation Profiling in Circulating Cell-Free DNA
Source: Cancers (Basel). 2026 Jun 23;18(13):2022. doi: 10.3390/cancers18132022 (PMC13359521; doi:10.3390/cancers18132022)

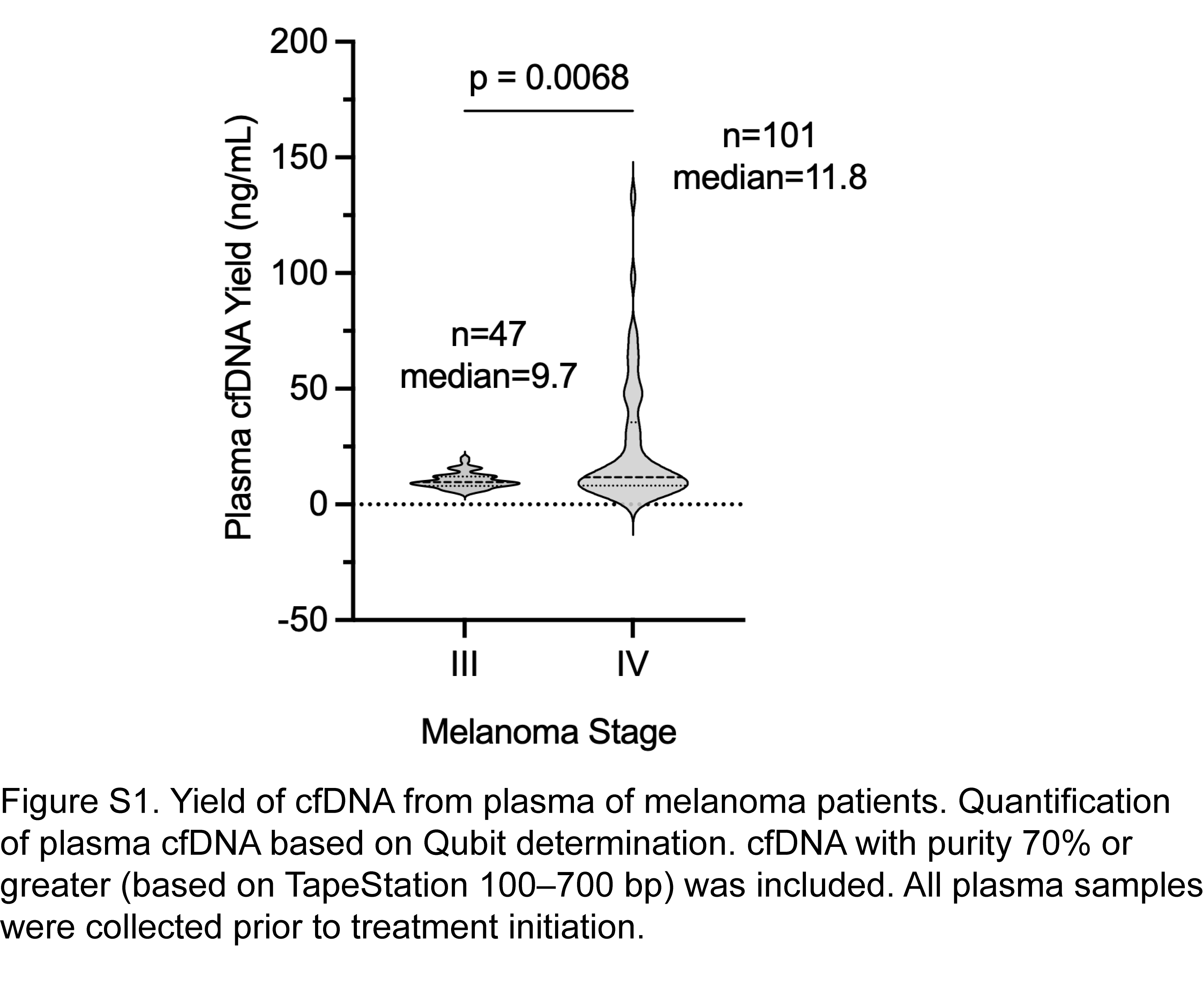

Supplement: Supplementary file 1 [file cancers-18-02022-s001.zip › Figure S1 Melanoma cfDNA yields.tif]

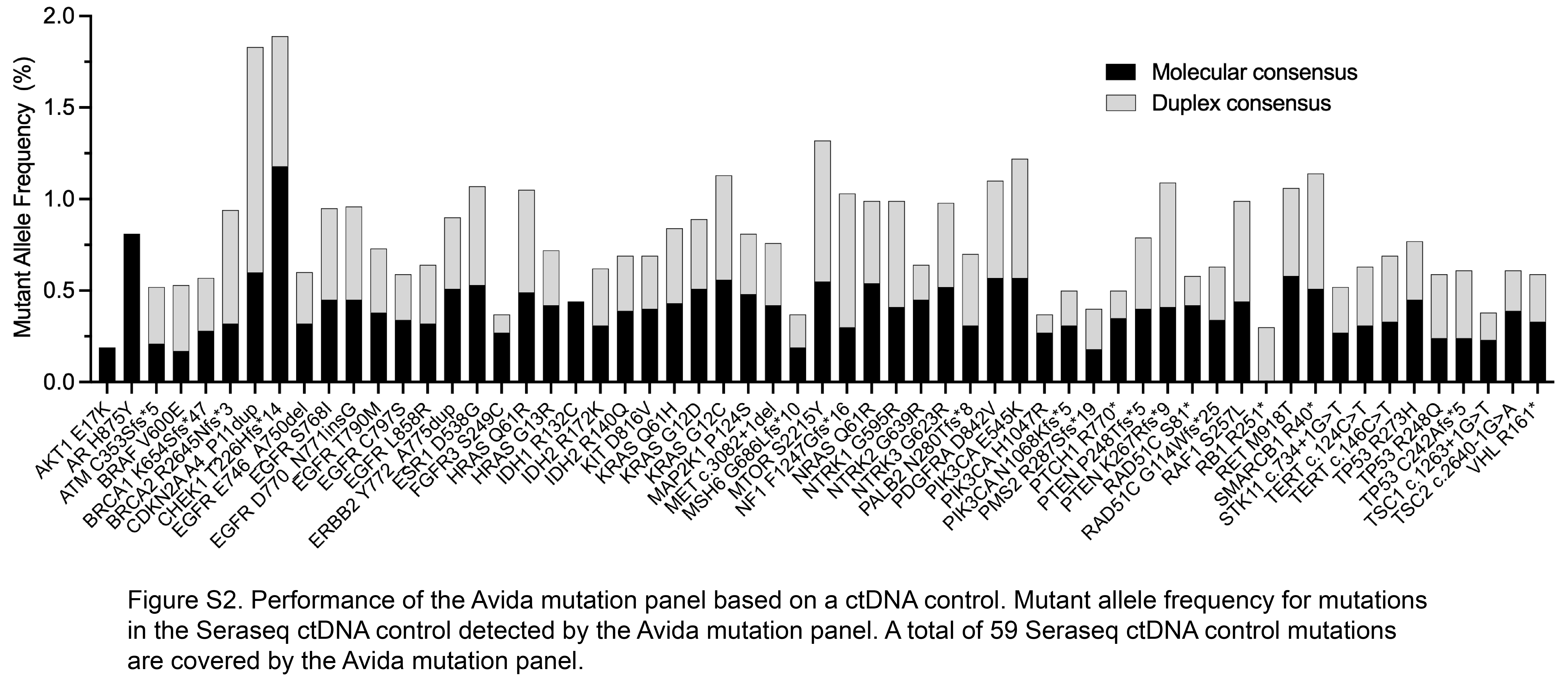

Supplement: Supplementary file 1 [file cancers-18-02022-s001.zip › Figure S2 Seraseq ctDNA control.tif]
